# Motivations for personal financial management: A Self-Determination Theory perspective

**DOI:** 10.3389/fpsyg.2022.977818

**Published:** 2022-09-20

**Authors:** Stefano I. Di Domenico, Richard M. Ryan, Emma L. Bradshaw, Jasper J. Duineveld

**Affiliations:** ^1^Department of Psychology, University of Toronto Scarborough, Toronto, ON, Canada; ^2^Institute for Positive Psychology and Education, Australian Catholic University, Sydney, NSW, Australia

**Keywords:** financial literacy, investing, motivation, personal finance, Self-Determination Theory

## Abstract

Financial knowledge and sound financial decision making are now broadly recognized to be important determinants of both personal and societal prosperity, but research has yet to examine how distinct qualities of motivation may be associated with the way people manage their money. In two studies we applied the framework of Self-Determination Theory (SDT) to examine people's autonomous (volitional) and controlled (pressured) motivation for understanding and managing their finances, as well as their amotivation (lack of motivation) for doing so, and the differential associations these motives have with financial knowledge and financial well-being. American participants (Study 1, *N* = 516; Study 2, *N* = 534) completed detailed demographic surveys and questionnaires assessing the financial variables of interest. As hypothesized, SDT's motivational constructs were associated with financial outcomes over and above participants' age, gender, income, household wealth, and educational attainment. Autonomous motivation was positively associated with a host of positive financial behaviors and characteristics (e.g., saving/investing and financial self-efficacy, well-being, and self-awareness). Controlled motivation was negatively associated with financial well-being. Amotivation was positively associated with overspending and negatively associated with financial self-efficacy and well-being. These findings support the relevance of SDT's framework in this domain and suggest that interventions aimed at promoting financial knowledge and wellness may benefit by adopting evidence-supported strategies for optimizing more autonomous motivations and addressing amotivations.

## Introduction

Personal finance is inescapable. From paying bills to budgeting, from retirement planning to filing taxes, and from paying the debt to saving and investing, people are continually tasked with managing their finances. Personal finance is also woven into other aspects of people's lives. Evaluating employment opportunities, planning vacations, and deciding where to live—buy or rent—are just a few examples of common goal-setting and decision-making activities that are shaped by the way people manage their money. When personal finances are not at the top of people's minds, news media are often there to put it back: Quantitative easing and inflation, tax cuts and unemployment, the stock market and interest rates, the prices of food, oil, and bitcoin—these and related topics flood the news cycle every day.

Given the pervasiveness and importance of financial decision-making, it is not surprising that policy advisors promote financial literacy (e.g., OECD, 2005; Lusardi and Mitchell, 2014; Lusardi, 2019). *Financial literacy* has been variously defined and measured (Huston, 2010; Cude, 2022). Some scholars treat financial literacy as a multidimensional construct that subsumes financial knowledge, numeracy, specific attitudes, and behaviors, as well as financial self-efficacy (Cude, 2022). Others use the terms financial literacy and *financial knowledge* interchangeably (Huston, 2010). Financial knowledge broadly refers to the knowledge and skills necessary for setting financial goals and making sound financial decisions. Financial knowledge entails understanding basic economic concepts (e.g., opportunity cost, inflation) and how financial products and services work (e.g., mortgages and interest rates). Because financial knowledge is common to most definitions and assessments of financial literacy (Lusardi and Mitchell, 2007; Cude, 2022), we here focus on financial knowledge.

People who are more financially knowledgeable and have other characteristics related to the broader financially literacy construct are more likely to avoid credit fees, have better credit scores, take measured investment risks, make better plans, set money aside for emergencies, and have greater financial well-being overall (Fernandes et al., 2014; Santini et al., 2019). Financial knowledge and literacy may also have broader societal import. According to the U.S. Financial Literacy and Education Commission (2020, p. 2), “Financial education is key to unlocking the foundations of economic opportunities and powering a strong and resilient economy. Americans must acquire financial skills and knowledge to fully participate in our dynamic economy.” In a related vein, Fornero et al. (2021) recently argued that, in our post-COVID world, financial and economic knowledge is especially important for fuller participation and decision-making in contemporary democratic processes.

Despite the importance of financial knowledge and sound money management for financial wellness, research has yet to systematically examine the motivations that drive people to learn about or better manage their finances. Illustratively, in a review of more than 500 peer-reviewed journal articles, Goyal and Kumar (2020) found three major research themes in the personal finance literature: the degree of financial literacy amongst distinct cohorts, and the influence of financial literacy on financial behavior, and the impact of financial education. Goyal and Kumar (2020) also reported that motivational constructs (e.g., risk tolerance, financial attitudes, and financial self-confidence) are diffusely represented in the extant literature, as either antecedent of financial literacy or as correlates and outcomes of financial education.

The absence of a comprehensive framework for researching people's motivations for personal finance is somewhat surprising since psychologists have long understood that people can behave in suboptimal ways, even when they “know better,” when they lack high-quality motivation (e.g., Sternberg, 2002). Understanding people's motivations for managing their finances is therefore of fundamental importance. Doing so promises to not only increase our understanding of why people manage their finances the way they do, but it could also help identify useful targets for enhancing financial education and advising.

In working toward understanding people's motivations for understanding and managing their finances, insights may be gleaned from the broader fields of motivational psychology and personality development, and in particular from Self-Determination Theory (SDT; Ryan and Deci, 2017). SDT is a longstanding framework for the study of human motivation. It differentiates autonomous (volitional) qualities of motivation from controlled (pressured) qualities of motivation, as well as amotivation (a lack of motivation), as different ways people may engage with their personal finances. Research across a variety of applied domains (e.g., work, education, healthcare) indicates that more autonomous forms of motivation are reliably associated with better performance and wellness outcomes (Ryan et al., 2021). We expect the same is true for the domain of personal finance. Importantly, SDT offers practical insights for enhancing people's motivation—a point to which we return in the Discussion. We, therefore, believe that SDT has much to offer basic and applied research in personal finance. The main purpose of the present article is to bridge research from personal finance and public advocacy with SDT to more comprehensively examine the motivations that underlie people's financial behaviors and well-being.

## A closer look at motivation: Self-Determination Theory

SDT makes the broad distinction between *autonomous* and *controlled* qualities of motivation. Autonomously motivated behaviors are enacted with a sense of volition. When a person feels autonomous, he or she concurs with or is willing to engage in particular activities. *Intrinsic motivation* is a type of autonomous motivation. It refers to people's engagement with activities that are driven by an interest or by the satisfaction that people experience during the enactment of the activities (e.g., an individual enjoys learning about financial “life hacks”). *Identified motivation* is another type of autonomous motivation that is evidenced when one consciously values activity and endorses its underlying goals (e.g., an individual builds an emergency savings fund upon assenting to its importance). In contrast to these autonomous motivations, SDT describes controlled motivations as those in which a person feels compelled or pressured to act. *Introjected motivation* is one type of controlled motivation. It describes behavior that is internally controlled by a sense that one “should” or “must” do something. Introjected motivations are often focused on obtaining approval from oneself or others and driven by shame, guilt, or anxiety (e.g., an individual adheres to a budget to avoid the personal shame of failing to do so). *External motivation* is another type of controlled motivation. It refers to activities that people do in order to obtain external rewards or avoid punishments. Such behaviors are dependent upon external contingencies; when the contingencies are removed, people lose their motivation to persist (e.g., an individual makes sure to pay their bills on time because they expect their spouse to be angry with them if they do not; when the spouse is not present, they are not concerned with their bills).

Both autonomous and controlled motivations describe the reasons why people act in particular ways (Ryan and Deci, 2017). In contrast, SDT characterizes *amotivation* as the lack of intention to act (i.e., a lack of motivation). When a person is amotivated, they either do not value a behavior or outcome (e.g., an individual does not care to learn about mutual funds because they do not perceive any utility in doing so), or believe that they are incapable of performing the activity (e.g., an individual does not invest in the stock market because they do not believe they have enough money to do so). The latter type of amotivation, which connotes feelings of hopelessness or a lack of efficacy, is typically associated with the poorest performance and affective outcomes.

SDT's model of motivation affords a differentiated perspective from which to examine how people manage their finances. Consider, for example, the varied motives one may have for monitoring their budget. An individual may be curious about their spending habits (intrinsic motivation), may find budgeting helpful for allocating funds to important purchases (identified motivation), may compulsively watch their spending to manage anxiety and guilt (introjected motivation) and may follow a budget because others pressure them to do so (external motivation), or may feel that following a budget is too hard or pointless (amotivation). How might these various motives impact one's financial behaviors and well-being?

The qualities of motivation specified by SDT have been researched across a variety of cultural settings, developmental epochs, and life domains (Ryan and Deci, 2017). Studies have consistently found that more autonomous (*vs*. controlled and amotivation) qualities of motivation are associated with greater behavioral persistence, deeper learning, and more effective performance, especially in activities that are difficult or that require long-term effort. Autonomous motivation is also reliably associated with more positive experiences and greater well-being. This literature is now voluminous (Ryan et al., 2019) so we cite a few examples for illustrative purposes.

In education, Domenico and Fournier (2015) found that university students who were more autonomously motivated for their schoolwork had higher grade-point averages, a statistical relationship that remained even when students' intelligence scores were taken into account. In healthcare, research shows that when people are autonomously motivated for health-related changes (e.g., smoking cessation, improving diet, exercising regularly, managing glucose), they are more successful and more likely to maintain positive changes over time (Ng et al., 2012). The benefits of autonomous motivation (*vs*. both controlled motivation and amotivation) in workplace settings have also been well documented (Deci et al., 2017) and supported meta-analytically (Slemp et al., 2018). In a study of school principals, Fernet et al. (2012) found that autonomous motivation was negatively related to work exhaustion but positively related to work commitment, whereas controlled motivation was positively related to exhaustion. SDT's model of motivation has also been applied to examining people's idiographic goal strivings. In this research, people list the personal goals that they are currently working toward and rate the motivation quality for each goal. Studies in this line show that more autonomous goals are associated with greater goal progress and psychological well-being (e.g., Holding et al., 2017). Given the research findings described above, we expect autonomous motivation to be associated with sound financial behaviors and greater financial well-being.

## Overview of the present research

The goal of the present research was to apply SDT's motivational framework to the domain of personal finance. Specifically, we aimed to adapt items from existing SDT questionnaires to assess respondents' quality of motivation for (a) monitoring their budgets, (b) paying bills in a timely manner, and (c) learning about new financial products and services. We hypothesized that the subscales measuring SDT's specific qualities of motivation—intrinsic, identified, introjected, external, and amotivation—would evidence internal reliability and a simplex-like pattern of inter-correlations, such that the largest correlations would appear along the main diagonal of the matrix (Ryan and Connell, 1989). This simplex-like association would be consistent with previous SDT studies and provide evidence of construct validity by suggesting that the varied motives are ordered along a continuum of relative autonomy (Howard et al., 2017; Donald et al., 2020).

We also hypothesized that the broad categories of autonomous motivation, controlled motivation, and amotivation would evidence a differentiated pattern of associations with respondents' degree of financial knowledge, financial behaviors (e.g., saving and investing, spending, financial self-awareness), financial self-efficacy, financial well-being, and general psychological well-being. Specifically, we hypothesized that autonomous motivation would be positively associated with financial knowledge, better financial behaviors, financial well-being, and general psychological well-being, and we expected controlled motivation and amotivation to evidence a suboptimal pattern of associations with these variables. These hypotheses were tested across two correlation studies that used diverse measures of financial behaviors and well-being.

## Study 1

### Study 1 method

#### Participants

The study received institutional review board approval from the Human Research Ethics Committee at the Australian Catholic University. Participants were 516 American adults recruited by Qualtrics, a professional panel company. We targeted adults between the ages of 30 and 50 years to ensure that the study participants would have meaningful amounts of experience managing their personal finances. Participants completed an online consent form before entering the study. Table 1 summarizes the general demographic information of the sample.

**Table 1:** Demographic information and frequency distributions.

	**Study 1**	**Study 2**
***N***	516	534
**% Female**	52	50
**Range in Age**	30–50	18–55
**Median Age**	39	39
**Standard Deviation of Age**	5.75	10.03
**Ethnicity**		
% White	83	71
% Black	8	14
% Other	9	15
**Current employment status**		
% employed full-time	59	46
% employed part-time	8	10
% self-employed	7	10
% unemployed	9	15
% retired	2	2
% sickness benefit (incapacity for work)	4	6
% other	11	11
**Personal income**		
% < $15,000	19	26
% $15,001–$25,000	13	16
% $25,001–$35,000	10	14
% $35,001–$50,000	9	16
% $50,001–$75,000	11	13
% $75,001–$100,000	12	6
% >$100,000	26	7
**Household Income**		
% < $15,000	10	19
% $15,001–$25,000	10	13
% $25,001–$35,000	8	13
% $35,001–$50,000	11	16
% $50,001–$75,000	15	16
% $75,001–$100,000	14	12
% >$100,000	32	11
**Educational Attainment**		
% some high school	2	5
% high school diploma or equivalent	15	28
% business or trade school	2	4
% some college	15	20
% Associate's degree	9	9
% Bachelor's degree	27	22
% some graduate or professional school	3	1
% graduate or professional degree	27	11

All percentages are rounded to the nearest whole number.

#### Measures

A list of the constructs in this study, along with the descriptive statistics of their measures, is provided in Table 2. We provide a brief description of each below.

**Table 2:** Study 1 means, standard deviations, and internal reliabilities for study questionnaires.

	***M***	**SD**	**α**
**Financial motivations**
Intrinsic motivation	4.97	1.23	0.87
Identified motivation	5.45	1.13	0.89
Introjected motivation	4.08	1.32	0.83
External motivation	3.19	1.14	0.78
Amotivation	2.64	1.29	0.86
**Financial knowledge**	2.57	1.59	0.60
**Personal finance behaviors**
Saving and investing	1.60	1.20	0.73
Overspending	1.91	0.83	–
Financial self-awareness	4.69	1.03	0.72
**Financial self-efficacy**	0.85	0.36	–
**Financial well-being**	0.00	0.79	0.85

Overspending was measured using a single Likert-type item. Financial Self-Efficacy was measured using a single binary-response item. Financial Well-Being was scored as the aggregate of five standardized items. *Chowdhry and Dholakia (2019; Study 3) used data from the 2015 National Financial Capability Study (NFCS; N = 27,564), in which the mean score across the six Financial Knowledge questions was M = 3.29 (SD = 1.67). The lower mean in the present sample may have been produced by sampling error given our relatively small sample. Alternatively, these mean differences may reflect genuine differences across the NFCS participants and the present community sample.

##### Income

Participants reported their *Personal Income* and *Household Incomes* for the 2020 calendar year by selecting one of nine categories that corresponded most closely with their annual earnings before the deduction of tax. The frequency distributions of these variables are presented in Table 1. The median household income bracket of our sample ($50, 001–75, 000) was consistent with the median income of $64, 994 for households between 2016 and 2020 (U.S. Census Bureau, 2022).

##### Household wealth

Participants reported their *Household Wealth*, which was described to them as “the amount of money you would have if you cashed in all of your household's assets—e.g., house, car, caravan, boat, jewelry, etc.—and paid off all the debts.” Participants selected one of 23 wealth brackets that most closely matched their household's wealth. The five most commonly selected wealth brackets were “less than $25,000,” “$25,001-$50,000,” “$50,001-$100,000,” “$100,001–$150,000,” and “$200,001–$250,001,” with 27.13%, 10.65%, 10.65%, 6.78% and 6.78% response proportions, respectively.

##### Financial knowledge

Participants were presented with a 6-question multiple-choice test that assessed their financial knowledge. These six questions were obtained from Lusardi and Mitchell's (2007) widely recognized measure of “financial literacy” (Lusardi and Mitchell, 2007). This measure has been adopted by the FINRA Foundation's National Financial Capability Studies (FINRA Investor Education Foundation, 2022) and has been used in other publications to validly assess respondents' financial knowledge (e.g., Chowdhry and Dholakia, 2019). These questions concerned financial concepts such as interest rates, differences between stocks and mutual funds, and basic economic concepts. A sample question is: “If interest rates rise, what will typically happen to bond prices?” Response options to this question were, “They will rise,” They will fall,” They will stay the same,” “There is no relationship between bond prices and the interest rate,” “I don't know,” and “Prefer not to answer.” Correct responses were coded as 1; others were coded as 0.

##### Saving and investing

Three items measured saving and investing behavior (Chowdhry and Dholakia, 2019): “Do you regularly contribute to a retirement account like a 401(k) or IRA?”; “Have you set aside emergency or rainy day funds that would cover your expenses for 3 months in case of sickness, job loss, economic downturn, or other emergencies?”; and “Not including retirement accounts, do you have any investments in bonds, mutual funds, or other investments?” “Yes” responses were coded with a value of 1 and the sum of “yes” responses was used to quantify participants' saving and investing.

##### Overspending

Spending patterns were measured with the question, “Over the past year, would you say your spending was less than, more than, or equal to your income?” Following Chowdhry and Dholakia (2019), “spending less than income” was coded as 1, “spending about equal to income” was coded as 2, and “spending more than income” was coded as 3.

##### Financial self-awareness

Nineteen items assessed financial self-awareness (Chowdhry and Dholakia, 2019). Participants were instructed to “indicate how well you know the exact value (dollar amount, interest rate, etc.)” for items like “Your total net worth,” “Your student loan debt,” and “The amount of money in your savings account(s).” They then indicated whether they knew the exact value, the approximate value, or did not know at all, coded respectively as 2, 1, and 0. Participants could also indicate if a particular question was not applicable to them. Scores were computed as the mean across applicable items.

##### Financial self-efficacy

This construct refers to feelings of confidence about successfully handling of financial matters (Cude, 2022). A single item, “I am good at dealing with day-to-day financial matters, such as checking accounts, credit and debit cards, and tracking expenses,” was used to assess participants' financial self-efficacy (Chowdhry and Dholakia, 2019). This item was presented with a binary response format: “Yes” (coded as 1; “No” coded as 0).

##### Financial well-being

Five items were standardized and aggregated to form a composite measure of financial well-being. These items were adapted from questionnaire packages used in FINRA Foundation's National Financial Capability Studies (FINRA Investor Education Foundation, 2022). The first item read, “Thinking of the next 10 years, how financially secure do you feel?” which participants rated on a 1–4 scale from “Insecure” to “Secure.” The second item read, “All things considered, how satisfied or dissatisfied are you with your standard of living?” The response scale for this item ranged from “Very Dissatisfied,” coded as 1, to “Very Satisfied,” coded as 7. The third item read, “Overall, thinking of your assets, debts, and savings, how satisfied are you with your current financial situation?” with the scale for this item ranging from 1, “Not at All Satisfied,” to 7, “Very Satisfied.” The fourth item read, “How often does it happen that you do not have enough money to afford the kind of food or clothing that you/your family should have?” rated on a scale from “Almost Never,” coded as 1, to “Almost Always,” coded as 5. This item was reverse scored. Finally, the fifth item read, “How much difficulty do you have in meeting the payment of bills?” with responses ranging from 1, “No Difficulty at All,” to 5, “A Lot of Difficulties”, also reverse scored.

##### Financial motivation

We developed a 45-item financial motivation questionnaire that was closely based on previous SDT-based assessments (Center for Self-Determination Theory, 2022). The questionnaire's instructions stated:

*People have different reasons for managing their personal finances. By personal finances, we mean spending, budgeting, saving, and investing. In other words, all the different ways you can use your money. Please rate each of the following statements in terms of how true it is for you using the scale below*.

Participants rated each of the statements using a Likert scale anchored from “Not at all True,” coded as 1, to “Very True,” coded as 7. To broadly represent the domain of personal finance, the statements on this questionnaire began with one of three stems: “I try to monitor my budget because…,” “I try to pay my bills on time because…,” and “I try to learn about financial products and services (e.g., different types of investments and savings accounts) because…” Each regulatory style was assessed with 3 items for each question stem, for a total of 9 items each for intrinsic, identified, introjected, external, and amotivation. The complete list of items is presented in Table 3. Table 4 shows that the different types of motivation evidenced an expected simplex-like pattern of associations, such that the largest correlations appeared along the main diagonal of the matrix (Ryan and Connell, 1989). Following previous SDT studies, autonomous regulation was scored as the average of intrinsic and identified motivation, and controlled regulation as the average of introjected and external motivation (Ryan and Deci, 2017).

**Table 3:** Financial motivation PLOC items.

***I try to monitor my budget because…***
1. I'm curious about tracking my own spending habits. (Intrinsic) 2. I enjoy understanding the ways I spend my money. (Intrinsic) 3. I enjoy planning how to spend my money. (Intrinsic) 4. It helps me spend my money in ways that I believe are most important. (Identified) 5. It helps me reach the important goals that I have for myself and my family. (Identified) 6. It helps me make sure I dedicate enough money for important purchases. (Identified) 7. I'd feel bad about myself if I didn't. (Introjection) 8. I'd feel like guilty if I didn't. (Introjection) 9. I don't want other people to think that I'm “too loose” with my money. (Introjection) 10. Others would get angry with me if I didn't. (External) 11. I don't want others to think I'm irresponsible. (External) 12. I have no choice; others force to. (External) 13. Actually, I gave up trying to keep track of a budget. (Amotivation) 14. Actually, I don't have a budget; I don't believe I really need one. (Amotivation) 15. Honestly, I don't have a budget; I don't know how to create one. (Amotivation)
***I try to pay my bills on time because…***
16. I enjoy the feeling of staying on top of my bills. (Intrinsic) 17. I feel a sense of accomplishment when I pay bills. (Intrinsic) 18. I'm interested to see how my bills change from month-to-month. (Intrinsic) 19. Paying bills on time helps me keep my spending well-organized. (Identified) 20. I believe in the importance of respectfully paying others for the services they provide me. (Identified) 21. Maintaining and improving my credit score will help me achieve the financial goals that are important to me. (Identified) 22. I'd be embarrassed if I didn't. (Introjection) 23. I'd feel like a “deadbeat” or “loser” if I didn't. (Introjection) 24. I'd get angry with myself if I didn't. (Introjection) 25. When I get a bill notification, it feels like a threat or warning. (External) 26. I feel threatened at the possibility of getting a bad credit score. (External) 27. Other people would get angry with me if I didn't. (External) 28. Actually, I easily forget to pay bills on time. (Amotivation) 29. Honestly, I don't really try; I just wait for notifications. (Amotivation). 30. I'm not sure; I sometimes wonder what would happen if I didn't pay. (Amotivation)
***I try to learn about financial products and services (e.g., different types of investments and savings accounts) because…***
31. I find these topics really interesting. (Intrinsic) 32. It's fun to learn new, and sometimes better, ways to manage my money. (Intrinsic) 33. I'm just naturally curious about these things. (Intrinsic) 34. Keeping up to date with such matters helps me plan for the future. (Identified) 35. Learning about such matters helps me make the best financial decisions. (Identified)
36. I think it's really useful to stay informed about such matters. (Identified) 37. I want others to think I'm knowledgeable about these types of things. (Introjection) 38. I don't want to sound like “an idiot” to others when such topics come up in conversation. (Introjection) 39. I'd feel guilty if I didn't keep up to date on such matters. (Introjection) 40. I'm worried that others (e.g., my employer, banks, credit card companies) might take advantage of me. (External) 41. I'm not a “sucker”; I stay informed to get the best for myself and my family. (External) 42. I feel like I'm forced to; others depend on me to know about finances. (External) 43. To be honest, I tried to learn but I gave up. (Amotivation) 44. Actually, I don't try to learn about these things; it's just not important to me. (Amotivation) 45. Actually, I kind of gave up trying to learn about these things; I usually get overwhelmed when I try. (Amotivation)

**Table 4:** Study 1 correlations among the five motivation subscales (below the diagonal) and 95% confidence intervals (above the diagonal).

	**Intrinsic**		**Identified**		**Introjected**		**External**	**Amotivation**
**Intrinsic**	–	[0.78,0.84]	[0.42,0.55]	[0.20,0.36]	[−0.13,0.04]
**Identified**		0.81^*^	–	[0.39,0.53]	[0.11,0.28]	[−0.32, −0.16]
**Introjected**		0.49^*^		0.46^*^	–	[0.55,0.67]	[0.13,0.29]
**External**		0.28^*^		0.20^*^		0.60^*^	–	[0.54,0.65]
**Amotivation**	–	0.04	–	0.24^*^		0.21^*^	0.59*	–

N = 516;

* p < 0.001.

#### Data analytic approach

We conducted two types of statistical analyses. First, we conducted correlational analyses to broadly examine the relationships between different qualities of financial motivation, personal finance variables, and well-being. We report the 95% confidence intervals of the correlations to compare the magnitudes of the different relationships; non-overlapping intervals allow for the inference that correlations differ in magnitude. We then conducted a hierarchical multiple regression procedure to examine the extent to which autonomous motivation, controlled motivation, and amotivation predict financial knowledge, saving and investing, spending, financial self-efficacy, and financial well-being after controlling for a host of factors from age to income. Partial *F*-tests were utilized to examine whether models explained significantly greater amounts of variance in the dependent variables.

### Study 1 results

#### Correlational analyses

Table 5 displays correlations between the financial motivations and all other variables. With respect to demographic characteristics, an inspection of the 95% confidence intervals indicated that autonomous motivation held a greater positive association with income, household wealth, and educational attainment than amotivation; autonomous and controlled motivation did not differ in their associations with these demographic characteristics. Age was negatively correlated with both controlled motivation and amotivation. These patterns of associations indicated the importance of controlling for demographic characteristics in subsequent analyses.

**Table 5:** Study 1 correlations of SDT financial motivations (*N* = 516).

	**Autonomous Motivation**	**Controlled Motivation**	**Amotivation**
Age^a^	−0.09 [−0.19,0.00]	−0.17 [−0.26, −0.08]	−0.19 [−0.27, −0.09]
Gender^b^	0.15 [0.06,0.23]	0.16 [0.07,0.24]	0.14 [0.05,0.22]
Income	0.31 [0.23,0.38]	0.26 [0.17,0.34]	0.13 [0.04,0.21]
Household wealth	0.26 [0.18,0.34]	0.19 [0.11,0.28]	0.07 [−0.01,0.16]
Educational attainment^c^	0.28 [0.20,0.36]	0.18 [0.10,0.27]	0.02 [−0.07,0.10]
Financial knowledge	0.12 [0.04,0.21]	0.04 [−0.05,0.12]	−0.15 [−0.23, −0.06]
Saving and Investing	0.38 [0.30,0.45]	0.24 [0.16,0.32]	0.05 [−0.04,0.14]
Overspending^d^	−0.05 [−0.14,0.04]	0.06 [−0.02,0.15]	0.26 [0.17,0.34]
Financial self-awareness^e^	0.39 [0.32,0.46]	0.11 [0.03,0.20]	−0.13 [−0.22, −0.05]
Financial self-efficacy	0.31 [0.23,0.38]	0.05 [−0.04,0.14]	−0.15 [−0.24, −0.07]
Financial well-being	0.40 [0.33,0.47]	0.07 [−0.02,0.15]	−0.16 [−0.24, −0.07]

a N = 437;

b N = 514;

c N = 515;

d N = 480;

e N = 514; Gender:−1 = Female, 1 = Male; 95% confidence intervals displayed beneath correlation coefficients in brackets.

The autonomous motivation was positively associated with financial knowledge, whereas amotivation was negatively related, and controlled motivation was unrelated. Interestingly, whereas autonomous and controlled motivation were both associated with saving and investing, amotivation was significantly correlated with spending exceeding earnings. And whereas autonomous motivation had significant positive associations with financial self-efficacy and financial well-being, amotivation was negatively correlated with these variables. Finally, all three types of motivation held significant associations with financial self-awareness, but autonomous motivation had the strongest positive association and amotivation was negatively associated.

#### Regression analyses

We used hierarchical regression to examine the incremental validity of autonomous motivation, controlled motivation, and amotivation in the prediction of financially relevant characteristics and outcomes over and above relevant participant demographics, namely, age, gender, income, household wealth, and educational attainment. Whereas these demographic characteristics were entered in Step 1, all three qualities of motivation were entered at Step 2. Partial *F*-tests were used to assess the incremental prediction of the overall models across Steps 1 and 2. In the unstandardized models, each predictor was group-mean centered so that the intercept could be interpreted as the unstandardized value of the outcome at the mean of all predictors. The variance inflation factors (VIFs) for each predictor in the models were computed to check for multicollinearity and each predictor's VIF was well below Cohen et al.'s (2003) rule of thumb of VIF <10, indicating that multicollinearity was not an issue. The residual plots of the regression models were visually inspected and no evidence of heteroscedasticity was found. The results of these regression analyses are summarized in Table 6.

**Table 6:** Study 1 regression analyses examining the incremental associations of SDT motivations on financial outcomes.

	**Step 1**								**Step 2**							
	**Age**		**Gender**		**H. Wealth**		**Inc**		**Edu. Att**.		**Aut. Mot**		**Con. Mot**		**Amot**.	
	***b*(SE)**	b^*^	***b*(SE)**	b^*^	***b*(SE)**	b^*^	***b*(SE)**	b^*^	***b*(SE)**	b^*^	***b*(SE)**	b^*^	***b*(SE)**	b^*^	***b*(SE)**	b^*^
**Financial Knowledge^a^**	0.06(0.01)	0.23^*^	0.03(0.08)	0.02	−0.01(0.02)	−0.05	0.00(0.06)	0	0.22(0.04)	0.29^*^	0.08(0.08)	0.05	0.06(0.09)	0.04	−0.20(0.07)	−0.16^*^
	*R*^2^ = 0.12, *F*_(5, 429)_ = 11.93, *p* < 0.001								*R*^2^ = 0.15, *F*_(8, 426)_ = 9.54, *p* < 0.001							
**Saving and Investing^a^**	−0.01(0.01)	−0.06	0.03(0.05)	0.02	0.02(0.01)	0.10^*^	0.29(0.04)	0.46^**^	0.10(0.03)	0.18^**^	0.18(0.05)	0.17^**^	−0.03(0.05)	−0.03	0.02(0.04)	0.02
	***R*^2^** **=** **0.48**, ***F*_(5, 429)_** **=** **80.69**, ***p*** **<** **0.001**								*R*^2^ = 0.51, *F*_(8, 426)_ = 54.89, *p* < 0.001							
**Over Spending^b^**	−0.02(0.01)	−0.15^**^	0.07(0.05)	0.08	0.00(0.01)	0	0.00(0.03)	0	−0.01(0.03)	−0.04	0.01(0.04)	0.02	−0.04(0.05)	−0.05	0.16(0.04)	0.25^**^
	*R*^2^ = 0.04, *F*_(5, 402)_ = 2.96, *p* = 0.012								*R*^2^ = 0.09, *F*_(8, 399)_ = 4.68, *p* < 0.001							
**Financial S-Aware^a^**	0.00(0.01)	0.09^‡^	0.01(0.04)	0.12^*^	0.02(0.01)	0.02	0.16(0.03)	0.05	0.01(0.02)	0.08	0.21(0.03)	0.41^**^	−0.15(0.04)	−0.03	−0.05(0.03)	−0.02
	*R*^2^ = 0.28, *F*_(5, 429)_ = 32.70, *p* < 0.001								*R*^2^ = 0.39, *F*_(8, 426)_ = 33.40, *p* < 0.001							
**Financial S-Efficacy^a^**	0.01(0.00)	0.11^*^	0.03(0.02)	0.10^‡^	0.00(0.00)	−0.02	0.04(0.01)	0.20^**^	0.01(0.01)	0.06	0.09(0.02)	0.27^**^	−0.03(0.02)	−0.08	−0.03(0.01)	−0.11^*^
	*R*^2^ = 0.08, *F*_(5, 429)_ = 7.87, *p* < 0.001								*R*^2^ = 0.17, *F*_(8, 426)_ = 11.14, *p* < 0.001							
**Financial W-B^a^**	0.00(0.01)	0.02	0.01(0.04)	0.01	0.02(0.01)	0.14^*^	0.16(0.03)	0.39^**^	0.01(0.02)	0.02	0.21(0.03)	0.30^**^	−0.15(0.04)	−0.20^**^	−0.05(0.03)	−0.09^‡^
	*R*^2^ = 0.28, *F*_(5, 429)_ = 32.70, *p* < 0.001								*R*^2^ = 0.39, *F*_(8, 426)_ = 33.40, *p* < 0.001							

a N = 435;

b N = 408;

‡ p < 0.10;

* p < 0.05;

** p < 0.001;

Gender: Female = −1, Male = 1; The effect size b(SE) is the unstandardized regression coefficients with mean-centered predictors; b* is the standardized regression coefficient. The p-values are rounded to the nearest thousandth. All other statistics are rounded to the nearest hundredth.

##### Financial knowledge

In Step 1, both age and educational attainment were significantly predictive of financial knowledge. In Step 2, amotivation evidenced a significant negative association with financial knowledge. This significant incremental effect was further supported with a partial *F*-test that formally compared the models at Step 1 and 2, Δ*R*^2^ = 0.03, *F*(3, 426) = 5.00, *p* = 0.002.

##### Saving and investing

In Step 1, household wealth, income, and educational attainment were significant positive predictors of saving and investing. In Step 2, autonomous motivation evinced a significant positive association with saving and investing. This significant incremental effect was further supported with a partial *F*-test that formally compared the models at Step 1 and 2, Δ*R*^2^ = 0.02, *F*_(3,426)_ = 6.61, *p* < 0.001.

##### Overspending

In Step 1, age held a significant negative relationship with overspending. In Step 2, amotivation was a significant positive predictor of spending. This significant incremental effect was supported with a partial *F*-test that formally compared the models at Step 1 and 2, Δ*R*^2^ = 0.04, *F*_(3,399)_ = 7.32, *p* < 0.001.

##### Financial self-efficacy

In Step 1, income held a significant positive relationship with financial self-efficacy. In Step 2, autonomous motivation was a significant positive predictor of financial self-efficacy and amotivation held a significant negative association with financial self-efficacy. These significant incremental effects were supported with a partial *F*-test that formally compared the models at Step 1 and 2, Δ*R*^2^ = 0.08, *F*_(3,426)_ = 4.79, *p* < 0.001.

##### Financial self-awareness

In Step 1, gender held a significant positive relationship with financial self-awareness. In Step 2, autonomous motivation was a significant positive predictor of financial self-awareness. This significant incremental effect was supported with a partial *F*-test that formally compared the models at Step 1 and 2, Δ*R*^2^ = 0.14, *F*_(3,424)_ = 24.98, *p* < 0.001.

##### Financial well-being

In Step 1, household wealth and income each held significant positive relationships with financial well-being. In Step 2, autonomous motivation evinced a significant positive association with financial well-being, whereas controlled motivation evinced a significant negative association with financial well-being. Amotivation was negatively associated with financial well-being, though this relationship was of marginal statistical significance. These significant incremental effects were supported with a partial *F*-test that formally compared the models at Step 1 and 2, Δ*R*^2^ = 0.10, *F*_(3,426)_ = 25.30, *p* = <0.001.

#### Study 1 brief discussion

The results of Study 1 were generally supportive of our hypotheses. Financial motives evidenced a simplex-like pattern of associations, consistent with previous research in SDT showing that these qualities of motivation are ordered along a continuum of relative autonomy (Howard et al., 2017). Regression analyses controlling for demographic characteristics showed that: (a) Autonomous motivation was positively associated with saving and investing, financial self-awareness, financial self-efficacy, and financial well-being; (b) Controlled motivation was negatively associated with financial well-being, and (c) Amotivation was negatively associated with financial knowledge and financial self-efficacy and positively associated with overspending.

## Study 2

In Study 2, we aimed to conceptually replicate and extend the findings from Study 1 using a different set of instruments to measure financial behaviors and well-being. Specifically, in addition to assessing the simplex-like pattern of associations among the different qualities of motivation and examining the differential associations that autonomous motivation, controlled motivation, and amotivation hold with financial knowledge, we used the Financial Health Network's *FinHealth Score*^®^ *Toolkit* (Financial Health Network, 2022a). This instrument is widely used by human resource professionals to assess and promote financial well-being among stakeholders. According to the Financial Health Network, “Financial health is a composite measurement of an individual's financial life. Unlike narrow metrics such as credit scores, financial health assesses whether people are spending, saving, borrowing, and planning in ways that will enable them to be resilient and pursue opportunities” (Financial Health Network, 2022b). The FinHealth Score^®^ Toolkit assesses respondents' financial health across four domains: Spending (e.g., spending less than income), saving (e.g., having sufficient liquid funds), borrowing (e.g., having manageable debt), and planning (e.g., being financially prepared). We also measured respondents' general psychological wellness by assessing their feelings of vitality and depletion (Ryan and Frederick, 1997) and their life satisfaction (Diener et al., 1985).

Hypotheses for Study 2 were preregistered (https://osf.io/qsker). We hypothesized that autonomous motivation would be positively associated with financial knowledge and indicators of healthy financial management whereas amotivation would be negatively associated with these outcomes, direct replication of Study 1. Previous work in SDT has shown that autonomous motivation is especially beneficial for endeavors that are complex, require sustained effort, and have a longer time horizon (Ryan and Deci, 2017). We accordingly hypothesized that autonomous motivation would evidence the strongest positive associations with the more deliberate and effortful aspects of financial health, namely, planning and borrowing. We expected amotivation to hold the strongest negative associations with these aspects of financial health. Finally, we hypothesized that whereas autonomous motivation would be associated with greater vitality, less depletion, and more life satisfaction, amotivation would have the opposite associations with these variables.

### Study 2 method

#### Participants

The study received institutional review board approval from the Human Research Ethics Committee at the Australian Catholic University. Participants were 534 American adults recruited by Qualtrics, a professional panel company. Participants completed an online consent form before entering the study. Table 1 summarizes the general demographic information of the sample.

#### Measures

A list of the assessments in this study, along with their descriptive statistics, is provided in Table 7. We provide a brief description of each below.

**Table 7:** Study 2 means, standard deviations, and internal reliabilities for study questionnaires.

	***M***	***SD***	**α**
**Financial motivations**			
Intrinsic motivation	4.75	1.29	0.88
Identified motivation	5.13	1.20	0.87
Introjected motivation	3.85	1.39	0.86
External motivation	3.22	1.20	0.78
Amotivation	2.72	1.29	0.86
Financial Knowledge^*^	2.58	1.63	0.62
**FinHealth ToolKit**^®^ **Scores**
Spend	68.16	25.41	–
Save	57.47	30.79	–
Borrow	63.59	26.74	–
Plan	58.19	27.49	–
**Vitality**	3.38	1.37	0.85
**Depletion**	2.78	1.24	0.85
**Life Satisfaction**	4.20	1.58	0.89

* Chowdhry and Dholakia (2019; Study 3) used data from the 2015 National Financial Capability Study (N = 27,564), in which the mean score across the six Financial Knowledge questions was M = 3.29 (SD = 1.67). Again, the lower mean in the present sample may be sampling error or may reflect a genuine differences in Financial Knowledge across the NFCS participants and our community sample.

##### Income

Participants reported their *Personal Income* and *Household Incomes* for the 2020 calendar year using the question used in Study 1. Again, the median household income bracket of our sample was $50,001–$75,000. Personal and household income were highly correlated (*r* = 0.73, *df* = 532, *p* < 0.0001) and were accordingly standardized and aggregated into a composite measure of income.

##### Household wealth

Participants also reported their *Household Wealth* using the questionnaire used in Study 1. The five most commonly selected wealth brackets were “ < $25,000,” “$25,001–$50,000,” “$50,001–$100,000,” “$100,001–$150,000,” and “$150,001–$200,000,” with 41.20%, 13.67%, 10.30%, 7.30%, and 3.93% response proportions, respectively. This distribution was similar to that reported in Study 1.

##### Financial knowledge

Participants completed the financial knowledge test that was used in Study 1.

##### Financial motivation

We administered the same 45-item financial motivation questionnaire developed in Study 1. Table 8 shows that the different types of motivation once again produced the expected simplex-like pattern of associations. The point estimates of these correlations were very similar to those obtained in Study 1. Autonomous regulation was again computed as the average of intrinsic and identified motivation and controlled regulation as the average of introjected and external motivation.

**Table 8:** Study 2 correlations among the five motivation subscales (below the diagonal) and 95% confidence intervals (above the diagonal).

	**Intrinsic**		**Identified**		**Introjected**		**External**	**Amotivation**
**Intrinsic**	–	[0.78,0.84]	[0.43,0.56]	[0.25,0.41]	[−0.09,0.00]
**Identified**		0.82^*^	–	[0.40,0.53]	[0.17,0.33]	[−0.31, −0.15]
**Introjected**		0.50^*^		0.47^*^	–	[0.55,0.67]	[0.15,0.31]
**External**		0.33^*^		0.25^*^		0.66^*^	–	[0.49,0.61]
**Amotivation**	–	0.09^*^	–	0.23^*^		0.23^*^	0.55^*^	–

N = 534;

* p < 0.001.

##### FinHealth Toolkit^®^

This 8-item questionnaire was developed by the Financial Health Network (Financial Health Network, 2022a) to assess four domains of financial health, each assessed with two items. Respondents are presented with a series of questions to which they may respond by selecting an answer that is most descriptive for them. The instrument's scoring manual assigns a specific score for each possible answer. Scores are computed as the mean across the two items for that domain. A sample item reads as follows: “How would you rate your credit score?” to which respondents and answer, “Excellent” (100 points), “Very good” (80 points), “Good” (60 points), “Fair” (40 points), “Poor” (0 points), and “I don't know” (0 points).

##### Psychological well-being

Subjective vitality was measured with Ryan and Frederick's (1997) 6-item scale. The scale's three positively worded items were used to assess feelings of Vitality proper (e.g., I have a lot of positive energy and initiative.”). The scale's three negatively worded items were used to assess feelings of Depletion (e.g., “I feel drained.”). The 5-item Satisfaction With Life Scale (Diener et al., 1985) asked participants to rate their agreement with each item on a 7-point Likert scale ranging from “Strongly Disagree” to “Strongly Agree.” A sample item is as follows: “In most ways, my life is close to my ideal.”

### Study 2 results

#### Correlational analyses

Consistent with Study 1 and with the broader SDT literature (Ryan and Deci, 2017), Table 8 displays that the different types of motivation once again evidenced the expected simplex-like pattern of associations, such that the largest correlations appeared along the main diagonal of the matrix (Ryan and Connell, 1989).

Table 9 displays correlations between the financial motivations and all other variables. The pattern of correlations between the financial motivations and the demographic variables was directionally consistent with the results obtained in Study 1 with a few notable exceptions. In this sample, the motivations were unrelated to gender, whereas in Study 1 females tended to score lower on all types of financial motivation. Furthermore, in the present sample, amotivation was negatively associated with income.

**Table 9:** Study 2 correlations of SDT financial motivations (*N* = 534).

	**Autonomous Motivation**	**Controlled Motivation**	**Amotivation**
Age	−0.14 [-0.22, −0.06]	−0.17 [−0.25, −0.09]	−0.06 [−0.14, 0.03]
Gender	−0.01 [−0.09, 0.08]	0.03 [−0.05, 0.12]	−0.06 [−0.14, 0.03]
Income	0.20 [0.11, 0.28]	0.07 [−0.01, 0.16]	−0.12 [−0.20, −0.03]
Household wealth	0.11 [0.02, 0.19]	0.00 [−0.08, 0.08]	−0.10 [−0.18, 0.05]
Educational attainment	0.09 [0.01, 0.18]	0.09 [0.01, 0.18]	−0.03 [−0.12, 0.10]
Financial knowledge	0.07 [−0.01, 0.16]	0.05 [−0.03, 0.14]	−0.20 [−0.27, −0.11]
Spend	0.18 [0.10, 0.27]	−0.01 [−0.09, 0.08]	−0.20 [−0.28, −0.12]
Save	0.30 [0.22, 0.37]	0.16 [0.07, 0.24]	0.02 [−0.06, 0.11]
Borrow	0.27 [0.19, 0.34]	0.11 [0.02, 0.19]	−0.12 [−0.20, −0.03]
Plan	0.37 [0.30, 0.45]	0.17 [0.09, 0.25]	−0.03 [−0.11, 0.05]
Vitality	0.45 [0.39, 0.52]	0.14 [0.05, 0.22]	−0.05 [−0.14, 0.03]
Depletion	−0.21 [−0.29, −0.13]	0.23 [0.14, 0.31]	0.38 [0.30, 0.45]
Life satisfaction	0.35 [0.27, 0.42]	0.14 [0.06, 0.23]	0.01 [−0.08, 0.09]

Gender: −1 = Female, 1 = Male; 95% confidence intervals displayed beneath correlation coefficients in brackets.

We hypothesized that autonomous motivation would again be positively associated with financial knowledge and that amotivation would again be negatively associated with financial knowledge. Autonomous motivation did not evidence this association. As a further exploratory analysis, we separately examined intrinsic motivation (i.e., acting out of enjoyment or interest) and identified motivation (i.e., acting consistently with abiding values or attributed importance). We found that whereas intrinsic motivation had no association with financial knowledge (*r* = 0.02, *df* = 532, *p* = 0.601), identified motivation did (*r* = 0.12, *df* = 532, *p* = 0.004). Amotivation held the expected negative association with financial knowledge.

Continuing with the correlations in Table 9, we further hypothesized that autonomous motivation would evidence the strongest positive associations with planning and borrowing, the ostensibly more deliberate and effortful aspects of financial health. We also expected amotivation to hold the strongest negative associations with these outcomes since deliberation and effort are depleted by amotivation. Contrary to our expectations, autonomous motivation held pronounced positive associations with each component of financial health. Moreover, amotivation held the strongest negative associations with participants' spend and borrow scores. The controlled motivation was positively correlated with three of the four components of financial health, though inspection of the correlation confidence intervals indicated that the magnitude of these correlations tended to be smaller than those obtained for autonomous motivation.

As predicted, autonomous motivation was associated with greater vitality, less depletion, and more life satisfaction. Amotivation was positively associated with depletion but did not evince significant associations with the other psychological well-being variables. We note that controlled motivation was positively associated with both vitality and life satisfaction, albeit, to a lesser degree than autonomous motivation, yet also positively associated with depletion. Though not hypothesized, we note that this latter association with depletion is consistent with controlled motivation as a suboptimal quality of motivation that is experienced as effortful compliance with external demands and internal pressures (Ryan and Deci, 2017).

#### Regression analyses

We again used hierarchical regression to examine the incremental validity of autonomous motivation, controlled motivation, and amotivation in the prediction of financial knowledge and well-being. Like Study 1, demographic characteristics were entered in Step 1 and the three qualities of motivation were entered in Step 2. Partial *F*-tests were used to assess the incremental prediction of the overall models across Steps 1 and 2. In the unstandardized models, each predictor was group-mean centered so that the intercept could be interpreted as the unstandardized value of the outcome at the mean of all predictors. The VIFs for each predictor in the models were checked and problematic multicollinearity was not found, nor did we find evidence of heteroscedasticity in the model residual plots. The results of these regression analyses are summarized in Table 10.

**Table 10:** Study 2 regression analyses examining the incremental associations of SDT motivations on financial outcomes.

	**Step 1**								**Step 2**							
	**Age**		**Gender**		**H. Wealth**		**Inc**		**Edu. Att**.		**Aut. Mot**		**Con. Mot**		**Amot**.	
	***b*(SE)**	b^*^	***b*(SE)**	b^*^	***b*(SE)**	b^*^	***b*(SE)**	b^*^	***b*(SE)**	b^*^	***b*(SE)**	b^*^	***b*(SE)**	b^*^	***b*(SE)**	b^*^
**Financial Knowledge**	0.01(0.01)	0.12^*^	0.38(0.07)	0.23^*^	0.01(0.02)	0.01	0.08(0.04)	0.12	0.19(0.03)	0.25^*^	−0.10(0.07)	−0.07	0.23(0.07)	0.17^*^	−0.30(0.06)	−0.24^*^
	***R*^2^** **=** **0.22**, ***F*_(5, 520)_** **=** **29.88**, ***p*** **<** **0.001**								***R*^2^** **=** **0.26**, ***F*_(8, 517)_** **=** **23.08**, ***p*** **<** **0.001**							
**Spend**	−0.07(0.11)	−0.03	0.65(1.11)	0.03	0.18(0.37)	0.24	2.36(0.72)	0.24^**^	−0.06(0.56)	−0.01	2.47(1.13)	0.12^*^	−0.36(1.22)	−0.02	−2.90(0.99)	−0.15^**^
	***R*^2^** **=** **0.07**, ***F*_(5, 520)_** **=** **7.67**, ***p*** **<** **0.001**								***R*^2^** **=** **0.11**, ***F*_(8, 517)_** **=** **7.89**, ***p*** **<** **0.001**							
**Save**	−0.34(0.13)	−0.11^**^	2.34(1.29)	0.08^‡^	0.47(0.43)	0.07	3.38(0.83)	0.30^**^	0.26(0.66)	0.02	7.12(1.30)	0.27^**^	−1.44(1.40)	−0.06	3.09(1.14)	0.13^**^
	***R*^2^** **=** **0.15**, ***F*_(5, 520)_** **=** **17.83**, ***p*** **<** **0.001**								***R*^2^** **=** **0.21**, ***F*_(8, 517)_** **=** **6.83**, ***p*** **<** **0.001**							
**Borrow**	−0.22(0.11)	−0.08^‡^	1.52(1.14)	0.06	0.46(0.39)	0.08	2.71(0.74)	0.26^**^	0.21(0.58)	0.02	4.20(1.16)	0.19^**^	0.61(1.26)	0.03	−1.33(1.02)	−0.06
	***R*^2^** **=** **0.11**, ***F*_(5, 520)_** **=** **13.33**, ***p*** **<** **0.001**								***R*^2^** **=** **0.16**, ***F*_(8, 517)_** **=** **12.00**, ***p*** **<** **0.001**							
**Plan**	−0.33(0.12)	−0.12^**^	1.80(1.19)	0.07	0.36(0.40)	0.06	2.71(0.77)	0.25^**^	−0.01(0.60)	0	8.51(1.16)	0.37^**^	−1.32(1.26)	−0.06	1.71(1.02)	0.08^‡^
	***R*^2^** **=** **0.10**, ***F*_(5, 520)_** **=** **11.52**, ***p*** **<** **0.001**								***R*^2^** **=** **0.20**, ***F*_(8, 517)_** **=** **16.53**, ***p*** **<** **0.001**							

N = 526, eight observations omitted because of missing values for gender;

‡ p <0.10;

* p <0.05;

** p <0.001;

Gender: Female = −1, Male = 1; The effect size b(SE) is the unstandardized regression coefficients with mean-centered predictors; b^*^ is the standardized regression coefficient. The p-values are rounded to the nearest thousandth. All other statistics are rounded to the nearest hundredth.

##### Financial knowledge

The results for financial knowledge mirrored those obtained in Study 1. In Step 1, both age and educational attainment were significantly predictive of financial knowledge. Inconsistent with the results of Study 1, gender was also significantly associated with financial knowledge in Step 1. In Step 2, amotivation once again evidenced a significant negative association with financial knowledge. Deviating from the results of Study 1, controlled motivation evidenced a positive association with financial knowledge in Step 2. We suspect this effect may have been obtained because of the associations between introjected and external motivation with amotivation (Table 8); with both autonomous motivation and amotivation partialled out of controlled motivation, the residual variance in controlled motivation may be an unstable predictor of financial knowledge. Nonetheless, the significant incremental effects found at Step 2 were supported with a partial *F*-test that formally compared the models at Step 1 and 2, Δ*R*^2^ = 0.04, *F*_(3,517)_ = 9.34, *p* < 0.001.

##### Spend

Income was the only significant predictor of spending scores at Step 1. In Step 2, autonomous motivation showed a significant and positive incremental association with respondents' spend scores, whereas amotivation had a significant and negative incremental association with spend scores. These significant incremental effects were supported with a partial *F*-test that compared the models at Step 1 and 2, Δ*R*^2^ = 0.04, *F*_(3,517)_ = 7.78, *p* < 0.001.

##### Save

Age was negatively associated with save scores at Step 1 whereas income was positively associated with saving scores at Step 1. In Step 2, autonomous motivation yielded a significant and positive incremental association with respondents' save scores. Unexpectedly, amotivation also held a significant and positive incremental association with saving scores. Like controlled motivation in the prediction of financial knowledge, we suspect that this regression result is an artifact of the correlations among the qualities of motivation and is not discussed further. These significant incremental effects were supported with a partial *F*-test that compared the models at Step 1 and 2, Δ*R*^2^ = 0.06, *F*_(3,517)_ = 13.09, *p* < 0.001.

##### Borrow

At Step 1, age held a marginally significant, negative association with borrow scores and income was positively associated with borrow scores. At Step 2, autonomous motivation evidenced an incrementally positively association with borrow scores, Δ*R*^2^ = 0.05, *F*_(3,517)_ = 8.79, *p* < 0.001.

##### Plan

Age held a significantly negative association with plan scores at Step 1, whereas income held a significantly positive association with this health measure. In Step 2, autonomous motivation was a significant and positive predictor of plan scores, whereas amotivation held a marginally significant, negative association with plan scores. A partial *F*-test supported the incremental difference between Steps 1 and 2, Δ*R*^2^ = 0.10, *F*_(3,517)_ = 22.50, *p* < 0.001.

#### Study 2 brief discussion

The correlational and regression analyses in Study 2 were largely consistent with the results obtained in Study 1. The financial motives again displayed the expected simplex-like pattern of associations (Howard et al., 2017). Autonomous motivation held consistently positive associations with most indicators of financial health whereas amotivation mostly held negative associations with these variables. Income was a consistent positive predictor of financial health.

## General discussion

How people manage their personal finances has become a salient concern for financial decision-making experts, economists, and policy advisors (OECD, 2005; Financial Literacy U.S. and Education Commission, 2020). The present research utilized SDT (Ryan and Deci, 2017) and its differentiated framework of human motivation to elucidate how the quality of people's motivation for managing their finances is related to people's financial knowledge, behaviors such as saving and investing, and financial well-being. Consistent with SDT, we expected that financial motives would array along a continuum of relative autonomy and that more autonomous forms of motivation would be associated with more effective financial management. We also predicted that controlled motivation, and especially amotivation, would be associated with less effective financial management and lower financial well-being. The results across two studies largely supported our hypotheses.

As expected, correlational analyses found that the financial motives were arranged into a simplex-like pattern of associations (Ryan and Connell, 1989), such that the largest correlations appeared along the main diagonal of the matrix (Tables 4, 8). The simplex-like associations suggest that these motives are systematically ordered along a continuum of relative autonomy as the theory predicts (Howard et al., 2017). Comparing autonomous and controlled qualities of motivation, the confidence intervals of correlational analyses also revealed that autonomous motivation was positively and more strongly associated with financial knowledge, financial self-efficacy, awareness, well-being, and overall psychological wellness. Amotivation was negatively associated with financial knowledge, financial self-efficacy, self-awareness, financial well-being, and psychological well-being. These results are broadly consistent with previous SDT studies showing that more autonomous qualities of motivation are predictive of enhanced quality of performance and well-being outcomes (Ryan and Deci, 2017).

Across both Studies 1 and 2, age evidenced a significant negative correlation with controlled motivation. It is possible that as people mature and take on more financial responsibilities, they become more accustomed to managing their personal finances and feel less pressured for doing so. Future studies could more closely examine this relationship. Longitudinal designs will be necessary for elucidating the possible developmental mechanisms mediating this negative association between age and controlled motivation.

Regression analyses further revealed that amotivation was negatively associated with financial knowledge over and above key demographic variables (i.e., age, gender, household wealth, annual income, educational attainment). This result highlights the importance of motivational factors in the development of financial knowledge. Specifically, it suggests that feelings of indifference and ineffectiveness are detrimental to the acquisition of financial knowledge. Regression analyses also examined whether autonomous motivation, controlled motivation, and amotivation were predictive of financial behaviors and well-being. Over and above key demographic variables, we found that autonomous motivation was generally positively associated with several financial well-being indicators and that amotivation was generally negatively associated with financial well-being indicators. Together, these results attest to the importance of motivation beyond demographic characteristics such as age, education, and income status.

The present results bear important implications for interventions that aim to improve people's financial knowledge and money management. Field studies and interventions using SDT across a variety of applied domains (e.g., education, work, healthcare) have shown that social-contextual factors can promote the development of more autonomous forms of motivation (Ryan and Deci, 2017). Specifically, teachers, managers, and advisors promote the development of autonomous motivation for specific activities by supporting the autonomy and competence of those they wish to motivate (Ng et al., 2012; Slemp et al., 2018). Autonomy support entails relating to target individuals by taking their perspective, providing a meaningful rationale for a recommended behavior, encouraging initiation, providing meaningful choices, and being responsive to their needs and concerns. Controlling contexts, in contrast, pressure people to think, feel, or behave in specific ways. In fact, SDT specifies an array of strategies to increase the internalization of new values, thus leading to more autonomous motivations and their positive consequences (e.g., Bradshaw et al., 2021).

The results also underscore the deleterious associations that amotivation has with both financial knowledge and management. Amotivations arise when people feel incapable or when they do not ascribe value to a particular activity or outcome (Ryan and Deci, 2017). Interventions aimed at addressing amotivation may accordingly focus on providing effecting-related feedback to foster a sense of competence. They may also focus on clarifying the possible benefits (and costs) of an activity so that individuals can find personally valued reasons for undertaking it. In light of the current findings, financial educators and service agents may benefit from using SDT as a framework for distinguishing different qualities of motivation and for addressing possible amotivations among their current and prospective clients to help them make fuller use of the financial services available to them.

Amotivations may also be sensitive to broader societal factors. Specifically, macroeconomic conditions (e.g., employment rate, inflation, wealth concentration) may represent contextual influences that can exacerbate or ameliorate amotivations (Di Domenico and Fournier, 2014; Ryan et al., 2019). For example, in the face of rampant asset inflation (e.g., fast rise in the costs of home ownership relative to increases in wages), individuals may feel increasingly frustrated and hopeless about achieving longer-term financial goals (e.g., saving for a home or retirement) and may be more likely disengage from effective financial practices. Future studies should examine this possibility.

Previous studies in SDT suggest that autonomous motivation, controlled motivation, and amotivation may have synergistic and compensatory interactions with other independent variables in the prediction of some consequential life outcomes (e.g., Di Domenico and Fournier, 2014). This may also be the case within the domain of personal finance, especially in light of the fact that some financial literacy scholars see financial literacy as a broad, multidimensional construct that includes relevant financial attitudes and behaviors (Huston, 2010; Cude, 2022). For example, individuals with greater financial knowledge may have greater financial well-being if they are also autonomously motivated to mobilize their knowledge in the management of their finances (a synergistic interaction). Alternatively, a high degree of financial self-efficacy may be a protective factor among those who feel amotivated, particularly when people do not value personal financial management (a compensatory interaction). Testing for statistically reliable interaction effects require suitably sized samples and the current samples were not collected to examine interaction effects. Future studies with adequately sized samples will be needed.

The current findings combined with previous applied studies in SDT encourage us to envision financial education programs and practices that use autonomy-supportive practices. Such a program would seem very useful. The 2018 National Financial Capability Study (Lin et al., 2019), which tested financial knowledge in a nationally representative sample of American Adults, found that financial knowledge continues to decline among Americans. Although 71% of respondents believed that they have a high level of financial knowledge, the study results indicated that financial knowledge dropped from 42% in 2009 to only 34% in 2018. The 2018 study found that only 7% of respondents obtained perfect test scores, that only 43% correctly answered a question about investment risk, and that only 26% were able to correctly identify the relationship between bond prices and interest rates. Given the importance of financial knowledge and active personal finance management for individuals to make sound decisions and fully participate in the economy (Financial Literacy U.S. and Education Commission, 2020), these results are alarming and signal a strong need for schools and financial institutions to improve the delivery of financial education. We believe these institutions can make use of SDT principles to enhance the quality of financial education and promote higher qualities of financial motivation.

### Limitations

The current study is not without its limitations. We measured respondents' qualities of motivation across three financial domains, namely, monitoring budgets, paying bills, and learning about new financial products and services. People's motivations for other important financial domains—e.g., retirement planning, paying taxes, purchasing insurance, and investing—were not directly captured by our assessment. Future research will be needed to examine if people's quality of motivation in other financial domains is similarly linked to important outcomes. Moreover, we developed the question stems for assessing financial motivations using the style of past SDT-based assessments (Center for Self-Determination Theory, 2022) but future studies might benefit by trying to refine our measure, for example, by trialing multiple items stems for each different financial domains.

We utilized self-report personal finance questionnaires used in previous studies. Future research should use more objective, behavioral measures to more precisely assess the associations between people's motivation quality and their financial behaviors (Campbell and Fiske, 1959). Another limitation concerns the demographic characteristics of the present samples. We recruited exclusively American participants. The sample was range-restricted in terms of age and the majority of them identified as Caucasian. Future studies should test whether the present findings generalize to distinct segments of the population, including people living in other nations and older adults. Also interesting would be assessing motivation in young adults, who may just be forming their habits and attitudes toward money management. Finally, we highlight the cross-sectional nature of these data. The present findings suggest that financial motivations are predictive of financial outcomes and well-being but longitudinal data are required to decisively evaluate these relationships.

## Conclusion

We believe that the present findings advance research on the determinants of personal financial management and demonstrate the potential utility of SDT as a framework for understanding the varied reasons why people enact or fail to enact sound financial practices. The results of the current study may have important implications for the design of effective interventions to enhance people's knowledge and efficacy in dealing with their money, especially given SDT's evidence-supported principles for enhancing autonomous motivation and its associated beneficial outcomes. Given the importance, and the apparent struggles, of people managing their personal finances, a focus on motivation may have potentially broad effects.

## Data availability statement

The datasets presented in this article are not readily available. We are open to sharing the dataset once we have finished publishing all relevant findings from it. Requests to access the datasets should be directed to s.didomenico@utoronto.ca.

## Ethics statement

The studies involving human participants were reviewed and approved by Human Research Ethics Committee at the Australian Catholic University. The patients/participants provided their written informed consent to participate in this study.

## Author contributions

SD and RR conceptualized the studies and wrote the manuscript. SD conducted the data analysis with inputs from all co-authors. EB and JD assisted with data collection and all aspects of manuscript preparation. All authors contributed to the article and approved the submitted version.

## Conflict of interest

The authors declare that the research was conducted in the absence of any commercial or financial relationships that could be construed as a potential conflict of interest.

## Publisher's note

All claims expressed in this article are solely those of the authors and do not necessarily represent those of their affiliated organizations, or those of the publisher, the editors and the reviewers. Any product that may be evaluated in this article, or claim that may be made by its manufacturer, is not guaranteed or endorsed by the publisher.
